# Phylogenetic Analyses and Biological Characterization of H9N2 Avian Influenza Virus Isolated from Chickens in China from 2022 to 2023

**DOI:** 10.3390/microorganisms14010037

**Published:** 2025-12-23

**Authors:** Yafen Song, Aoyang Yan, Shengyao Song, Hongxuan Gong, Ling Chen, Bofan Fu, Min Zhang, Jie Zhang, Ji Liu, Yitong Guo, Guanlong Xu, Chenghuai Yang, Qianyi Zhang

**Affiliations:** 1 China Institute of Veterinary Drug Control, Beijing 100081, China; songyafen1@126.com (Y.S.);; 2 College of Animal Science and Technology, Shihezi University, Shihezi 832000, China; 3 College of Veterinary Medicine, Qingdao Agricultural University, Qingdao 266109, China; 4 College of Veterinary Medicine, Gansu Agricultural University, Lanzhou 730000, China

**Keywords:** avian influenza virus, H9N2, evolution, reassortment, pathogenicity, humoral immunity

## Abstract

The continued diversification of the H9N2 avian influenza virus (AIV) into multiple antigenically and phylogenetically distinct lineages is promoting the emergence of strains with pandemic potential. Constant monitoring of the genetic evolution and changes in biological characteristics of the H9N2 viruses is therefore essential. In this study, we analyzed the genetic evolution of the H9N2 viruses isolated from poultry farms between 2022 and 2023 and evaluated their pathogenicity in chickens and mice. The *HA* genes of all ten isolates belonged to the h9.4.2.5 lineage, which is currently the predominant evolutionary lineage in China. Yet, their *HA* genes further divided into distinct subbranches within the h9.4.2.5 lineage. The *NA* genes of these viruses shared high homology with the prevalent H9N2 AIVs in recent years. However, these viruses were located in different evolutionary groups. Notably, the internal genes showed close relationships with those of recent H3, H6, and H9 subtype AIVs, suggesting active reassortment events among co-circulating viruses. Pathogenicity assessment in mice and chickens demonstrated divergent virulence between two representative isolates, FS22 and JM14, which clustered into different h9.4.2.5 subbranches. FS22 exhibited more efficient and prolonged replication in the lungs and turbinates of mice compared to JM14. Both viruses replicated efficiently in the lungs, kidneys, and trachea of chickens at 3 days post-infection (DPI), but differed in their horizontal transmission potential. Particularly, inoculated and contacted chickens all produced high antibody levels from the 5 DPI until the end of the experiment, and peak antibody titers for both viruses occurred at 7 DPI. These findings underscored the continuous evolution ofH9N2 AIV enhanced its genetic and phenotypic diversity, leading it to pose a threat to public health. Thus, continuous surveillance in poultry farms is necessary.

## 1. Introduction

The H9N2 AIV was first isolated from turkeys in Wisconsin, USA, in 1966 and had become panzootic by the mid-1980s across Asia, the Middle East, Europe, and Africa [1], being one of the most widespread AIVs in poultry globally. Recent studies confirm H9N2 AIV ongoing endemicity and evolution, driving both economic losses and acting as a key genetic contributor to emerging viruses [2,3,4,5]. Unlike highly pathogenic H5 and H7 AIVs, H9N2 is classified as a low pathogenic avian influenza virus (LPAIV). It typically induces clinical signs ranging from mild to severe respiratory disease in domestic poultry, accompanied by reduced egg production and, in certain instances, mortality rates of up to 20%. However, co-infections with other respiratory pathogens [6,7], such as infectious bronchitis virus, *Mycoplasma gallisepticum*, and *Escherichia coli*, can markedly exacerbate the severity of H9N2 infection, leading to significantly higher mortality and substantial economic losses in the poultry industry.

Of particular concern is the ability of H9N2 AIV to cross species barriers and infect mammals, including humans, directly without the requirement for intermediate hosts. Since December 2015, the World Health Organization (WHO) has documented 149 human infection cases in the Western Pacific Region, including two fatalities, with 146 of these cases reported from China [8]. Furthermore, transmission of H9N2 AIV from poultry to pigs and dogs has been confirmed [9,10]. Additionally, H9N2 AIV serves as a crucial genetic reservoir, donating its internal genes to various novel AIVs that have infected humans, including H3N8, H5N1, H5N6, H7N9, and H10N8 [11,12,13]. Consequently, H9N2 AIV represents a persistent and significant threat to public health.

In China, H9N2 AIV was first identified in Guangdong province and has since been detected nationwide, establishing itself as the dominant influenza subtype in poultry. Vaccination has been a key tool in efforts to combat H9N2 AIV since the late 1990s, with at least twenty different commercial inactivated vaccines currently in use [14]. However, akin to other RNA viruses, H9N2 AIV undergoes rapid evolution via antigenic drift and shift, facilitating the frequent emergence of novel variants and subtypes capable of causing epidemics, zoonotic spillover, and potential pandemics. In China, the continuous antigenic drift of H9N2 AIV under vaccine immunization pressure has led to the selection of immune escape variants, compromising the effectiveness of existing vaccines and failing to provide solid protection against these H9N2 antigenic variants. Therefore, sustained surveillance of H9N2 AIV is imperative to guide the development of targeted and effective control strategies.

## 2. Materials and Methods

### 2.1. Ethical Approval

All experiments were conducted in biosafety level 2 (BSL2) laboratories of the China Institute of Veterinary Drug Control (IVDC). The handling of chickens and mice were performed in accordance with the approved guidelines of the Experimental Animal Administration and Ethics Committee of the IVDC [Permit Number 202400329; 13 October 2024].

### 2.2. Viruses

From 2022 to 2023, a total of 310 oropharyngeal and cloacal swab samples were collected from poultry farms in the Shandong, Jiangsu, and Guangdong provinces of China, and 10 H9N2 AIV strains were isolated from healthy chickens (Table S1). These H9N2 viruses were identified by reverse-transcription polymerase-chain reaction (RT-PCR), hemagglutination test (HA), and hemagglutination inhibition (HI) test in accordance with the Chinese National Standard GB/T 18936-2020 [15]. Briefly, the samples were suspended in phosphate-buffered saline (PBS) containing 5000 IU/mL penicillin and 5 mg/mL streptomycin. After centrifugation at 3000× *g* for 10 min at 4 °C, the supernatants were screened for H9N2 subtype AIV by RT-PCR. The RT-PCR-positive samples were propagated in the allantoic cavities of 9-day-old SPF chicken embryos for virus isolation. The identified isolates were subsequently purified by five rounds of limiting dilution in 9-day-old SPF embryos.

### 2.3. Phylogenetic and Sequence Analysis

The viral RNA was extracted from allantoic fluid using TaKaRa MiniBEST Viral RNA/DNA Extraction Kit Ver.5.0 (Takara Biomedical Technology, Beijing, China), and the genes coding sequences were amplified by RT-PCR using PrimeScript™ One Step RT-PCR Kit Ver.2 (Dye Plus) (TaKaRa, Beijing, China) with specific primers [16]. The amplified products were sent to the BGI Tech Solutions (Beijing liuhe) Co., Ltd., Beijing, China for sequencing. The sequences were assembled and analyzed by Lasergene 7.1 (DNASTAR, Madison, WI, USA). Phylogenetic trees were generated by the distance-based neighbor-joining method using software MEGA 4.0 (Sinauer Associates, Inc., Sunderland, MA, USA). The reliability of the tree was assessed by bootstrap analysis with 1000 replicates. The sequences generated in this study have been deposited in the GenBank database under the accession numbers listed in Table S1.

### 2.4. Animal Experiment

The 3-week-old white leghorn specific-pathogen-free (SPF) chickens and 9–10-day-old SPF embryonated chicken eggs were purchased from Beijing Boehringer Ingelheim Vital Biotechnology Co., Ltd., Beijing, China. Five-week-old SPF female BALB/c mice were purchased from the Beijing Vital River Laboratory Animal Technology Co., Ltd., China (Beijing, China). The chickens and mice were placed in the biological safety isolator and independent ventilation cages, respectively. Humane endpoints were observed and utilized over the entire experimental period.

Based on the phylogenetic analysis of the HA gene, two representative H9N2 viruses (JM14 and FS22) were selected for animal studies, following established protocols [17,18]. In the chicken study, 13 3-week-old SPF white leghorn chickens were inoculated intranasally with 0.2 mL of allantoic fluid containing 10^6^ EID_50_ of JM14 or FS22 virus. Five additional chickens, inoculated with 0.2 mL of PBS, served as contact controls and were co-housed with the infected groups. At 3 days post-infection (DPI), three chickens from each group were euthanized to assess viral replication in the heart, liver, spleen, lung, kidney, brain, and trachea. The remaining chickens were monitored for clinical signs for 14 days. During the experiments, chickens were euthanized when they could not be either unable to eat or drink. Oropharyngeal and cloacal swabs were collected at 1, 3, 5, 7, and 9 DPI, suspended in 1 mL of PBS, and tested by RT-PCR using the primers H9-273U-TGTGTCTTACGATGGGACAAGCA and H9-273L-TTGACAAGAGGCCTTGGTCCTAT from the Chinese national standard GB/T 18936-2020. Tissue samples (1 g/tissue) were homogenized in 1 mL of PBS containing antibiotics (1000 U/mL penicillin and 1000 µg/mL streptomycin). After centrifugation, supernatants were serially diluted and inoculated into the allantoic cavity of 9-day-old SPF chicken eggs (0.1 mL /egg). Allantoic fluids were harvested and tested for hemagglutination activity after incubation at 37 °C for 120 h. Virus titers were calculated by the Reed-Muench method. Blood samples were collected from both infected and contact chickens at 3, 5, 7, 9, 11, and 13 DPI, and serum antibody responses were evaluated by HI test. HI antibody titers of chicken homologous and heterologous antisera were determined by HI assay using 4 HAU of the JM14 antigen and FS22 antigen, respectively. Data shown were the mean antibody titers (log_2_) ± standard deviation.

In the mouse experiment, 33 5-week-old female SPF BALB/c mice were randomly divided into three groups. Two groups were lightly anesthetized with CO_2_ and inoculated intranasally with 10^6^ EID_50_ of JM14 or FS22 virus in 0.05 mL, while the control group received 0.05 mL of PBS. At 3 and 5 DPI, 3 mice per group were euthanized, and viral loads in the heart, livers, spleen, lung, kidney, brain, and nasal turbinate were determined by titrating tissue supernatant in 9-day-old SPF chicken embryos. The remaining mice were observed for 14 days to monitor clinical signs, weight changes, and mortality, the body weight of mice was lost more than 25% were euthanized during the experiments.

### 2.5. Statistical Analysis

Statistical analyses were used by the GraphPad Prism 10 software (GraphPad Software Inc., San Diego, CA, USA). Statistical analyses were performed by using a two-way ANOVA. *P* values of <0.05 were considered significant (* *p*  <  0.05; ** *p*  <  0.01, *** *p*  <  0.001, **** *p*  <  0.0001).

## 3. Results

### 3.1. Phylogenetic Analysis

**Figure 1:**
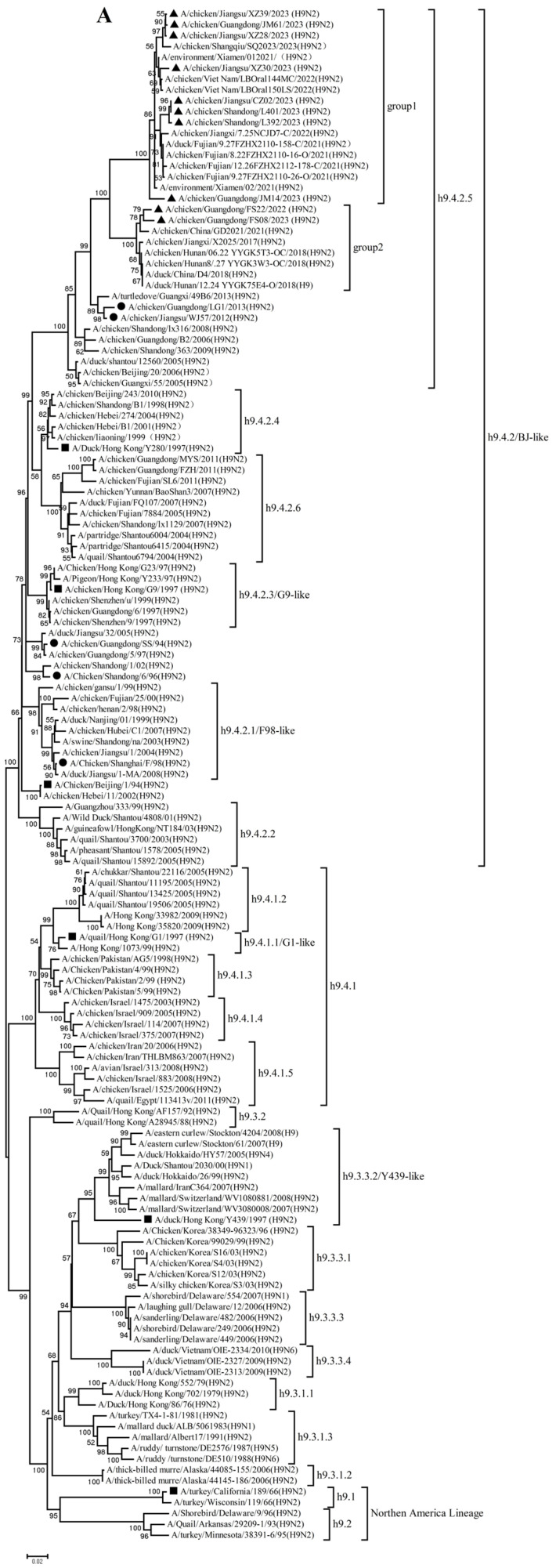

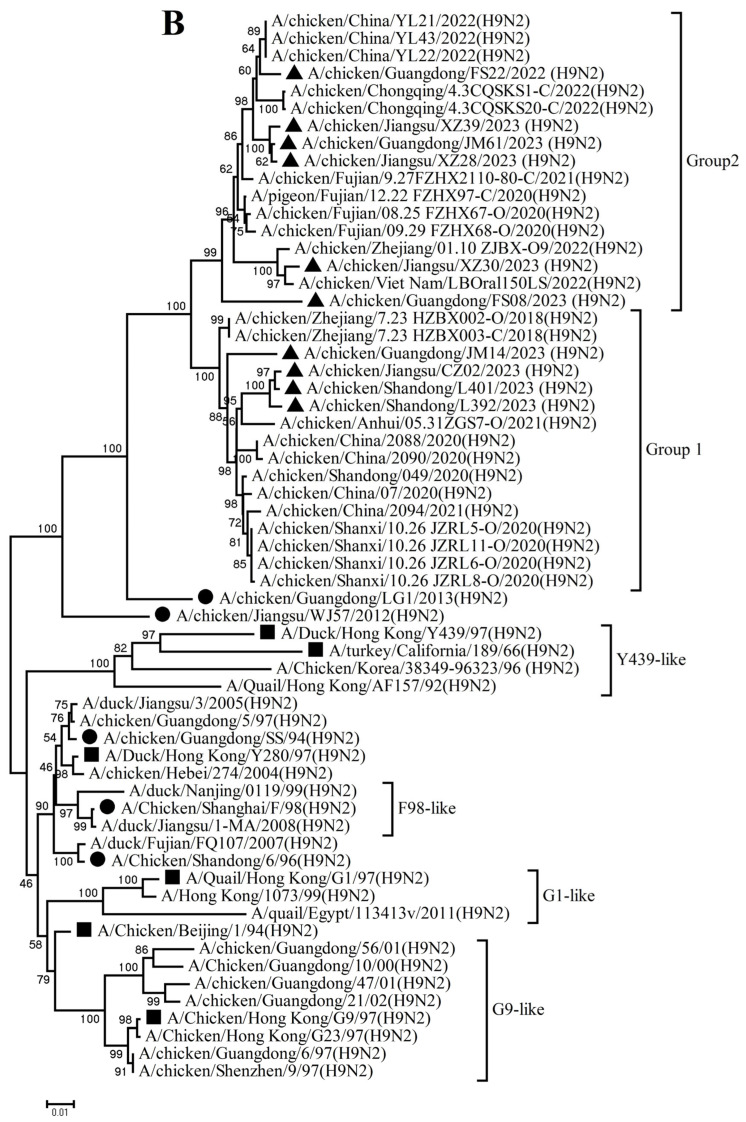
Phylogenetic analysis of hemagglutinin (HA) (**A**) and neuraminidase (NA)(**B**) genes. The viruses in this study, vaccine strains and reference viruses in the phylogenetic tree were marked with black triangles, circles, and squares, respectively.

To better understand the evolutionary trend and divergence of the new H9N2 isolates, phylogenetic analysis was carried out for the eight gene segments. Phylogenetic analysis found that the HA genes were divided into four evolutionary branches (Figure 1A). The predominant lineage, h9.4.2, was further divided into six sub-lineages (h9.4.2.1–h9.4.2.6). All 10 H9N2 isolates from 2022–2023 clustered within the h9.4.2.5 sub-lineage, which is currently the most prevalent lineage in China. These HA genes shared 91.1–99.9% nucleotide and 92.2–99.8% amino acid identity and segregated into two distinct groups: Group 1 comprised JM14, JM61, CZ02, XZ28, XZ30, XZ39, L392, and L401, while Group 2 included FS22 and FS08 (Figure 1A, Table S2). Notably, the HA phylogenetic tree revealed a considerable genetic distance between our isolates and currently used vaccine strains in China (87.1–93.5% nucleotide identity), as well as reference viruses from different lineages dating to the 1990s (78.4–88.3% nucleotide identity).

Analysis of the NA genes indicated that these H9N2 isolates had high homology with recently circulating H9N2 viruses in Asia, particularly China. These NA genes further diverged into two subgroups: 4 isolates in Group 1 shared 96.2–99.7% nucleotide identity, and 6 isolates in Group 2 shared 94.7–99.7% nucleotide identity (Figure 1B, Table S3). These NA genes shared 87.8–93.6% and 82.1–89.6% nucleotide identity with the vaccine and reference strains, respectively.

**Figure 2:**
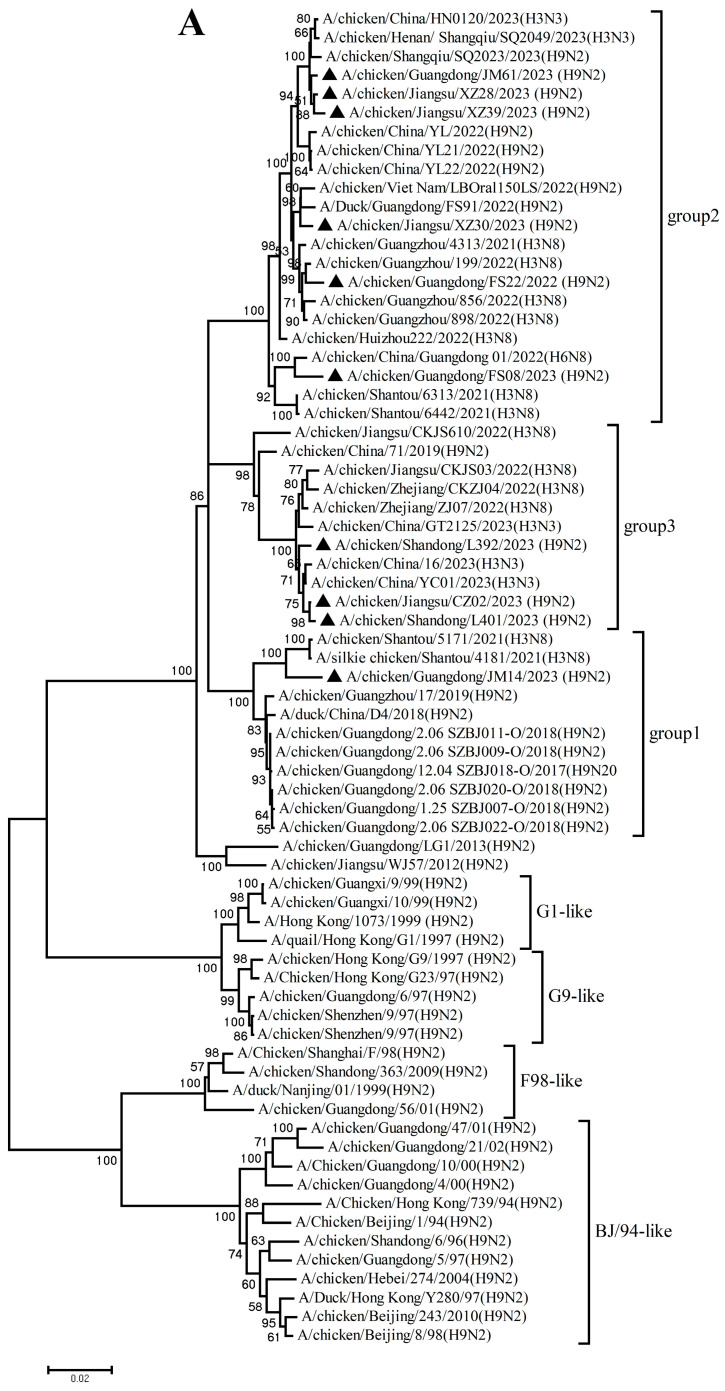

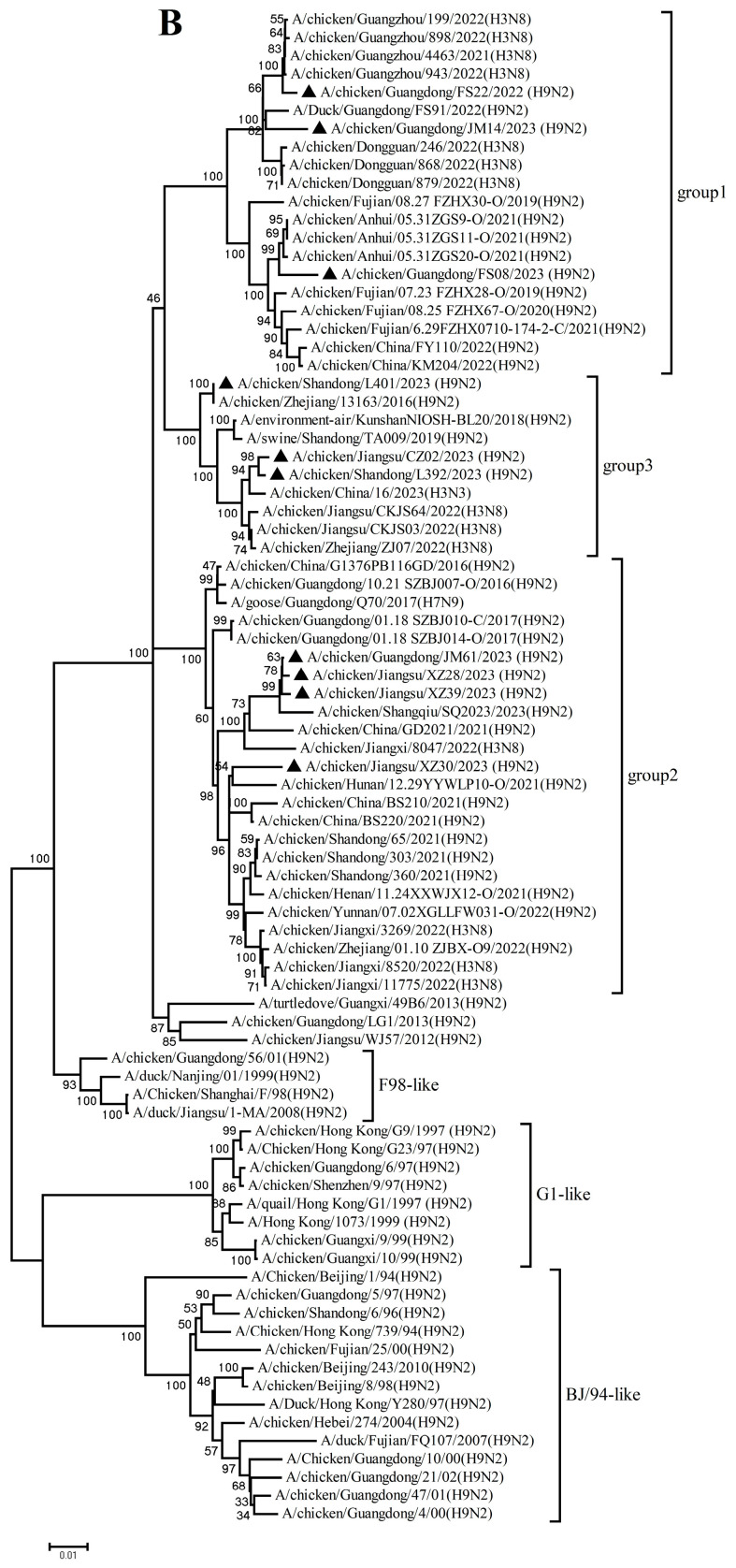

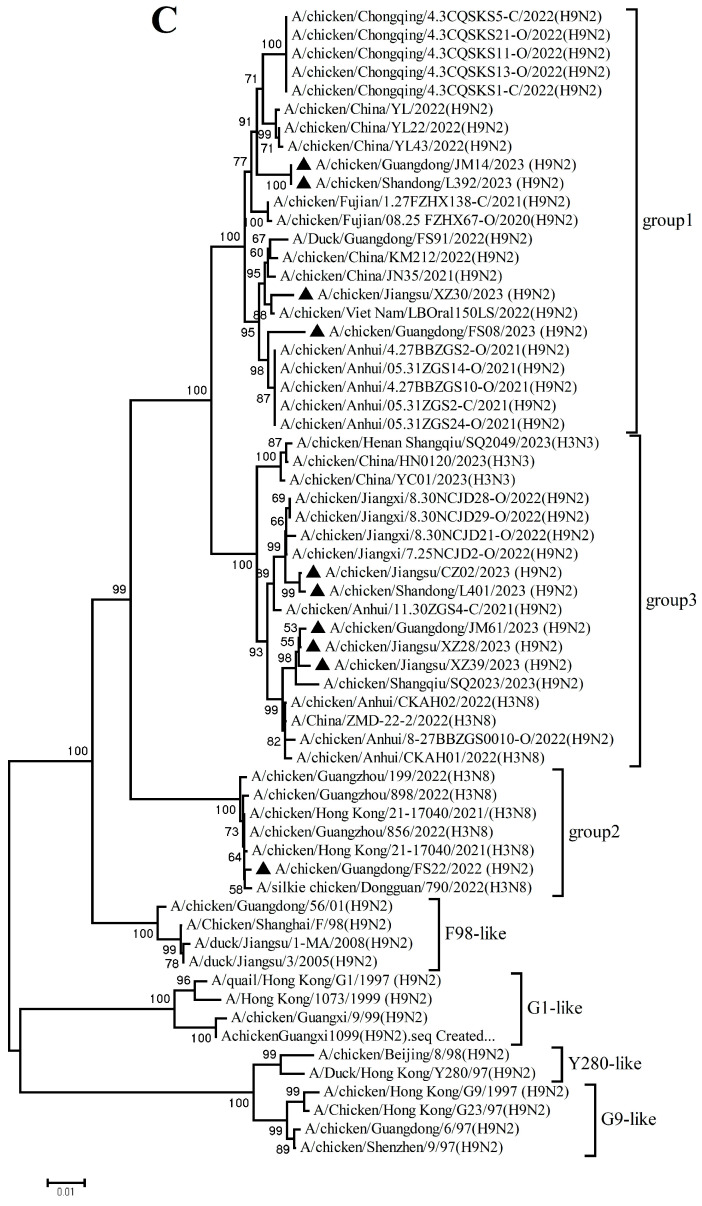

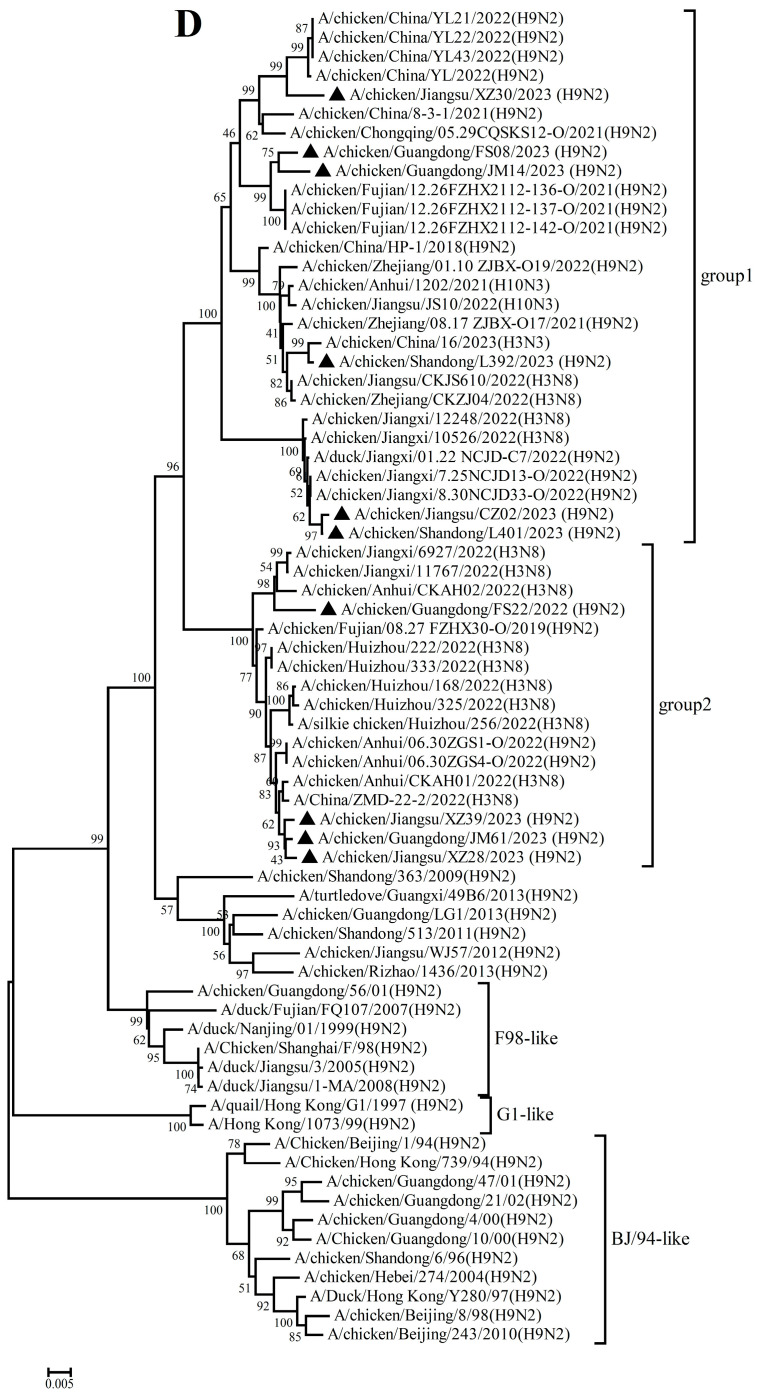

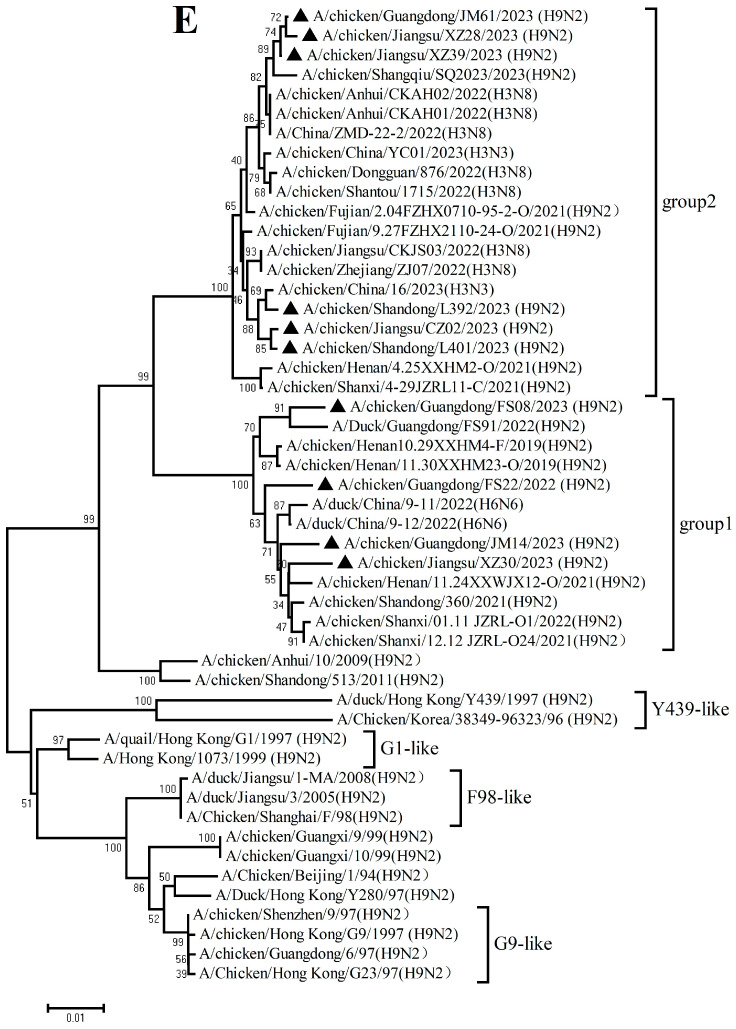

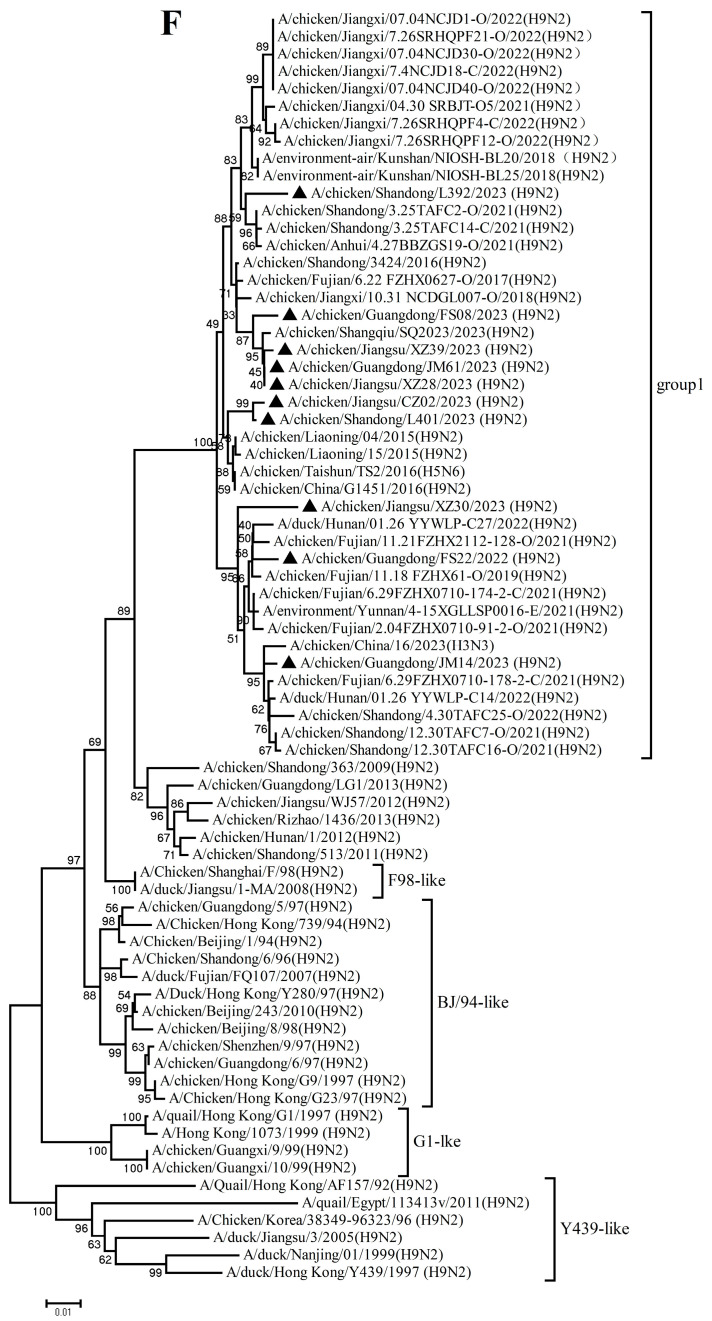
Phylogenetic analysis of six internal genes. polymerase basic subunit 2 (PB2) (**A**),  polymerase basic subunit 1 (PB1)(**B**), polymerase acidic subunit (PA) (**C**),nucleoprotein (NP) (**D**), matrix (M) (**E**),and nonstructural (NS) (**F**) genes. The viruses in this study were marked with a black triangle.

The PB2, PB1, PA, NP, M, and NS genes sharing 92.8–99.7%, 92.3–99.8%, 93.7–99.9%, 94.1–99.9%, 94.5–99.8%, and 96.0–100% nucleotide identity among these new H9N2 isolates, respectively. These six internal gene segments of these viruses showed diverse evolutionary patterns. The PB2, PB1, and NP genes of the isolates fell within a gene pool shared with H3 and H9 AIVs but were distributed across different groups (Figure 2A,B,D). The PA genes segregated into three major groups (Figure 2C): Group 1 (JM14, L392, XZ30, FS08) clustered exclusively with H9 AIVs; Group 2 (FS22) clustered with H3 AIVs; and Group 3 (CZ02, L401, JM61, XZ28, XZ39) formed a mixed clade with H3 and H9 AIVs. In the M gene tree (Figure 2E), four isolates grouped with H6 and H9 AIVs (Group 1), while the remaining six clustered with H3 and H9 AIVs (Group 2). In contrast, the NS genes of all isolates formed a homogeneous cluster with H9N2 AIVs recently prevalent in China. The results of the above analysis were also reflected in Table S4, which showed the highest nucleotide homology of each gene of ten H9N2 viruses determined by BLAST (https://ngdc.cncb.ac.cn/blast/home, accessed on 18 December 2025) search in the GenBank.

Collectively, these findings demonstrate that the h9.4.2.5 lineage of H9N2 AIVs had undergone continuous evolution in domestic poultry, driven by multiple independent reassortment events with co-circulating H3, H6, and H9 subtype AIVs.

### 3.2. Molecular Characterization

To elucidate the genetic characteristics of the newly isolated H9N2 AIVs, the deduced amino acid sequences were aligned and compared with vaccine strains and representative H9N2 isolates. Specific residues previously reported to influence antigenicity, pathogenesis, host tropism, and drug resistance were also analyzed.

As shown in Tables S5–S7, all viruses contained the amino acid motif PSRSSR↓GLF at the cleavage site of the HA protein, indicative of low pathogenicity and consistent with the hallmark feature of predominant H9N2 strains circulating in China since 2013 [19]. With the exception of FS08, all isolates carried L at position 226 (H3 numbering), which is associated with preferential binding to human-type receptors. All viruses retained the avian-like receptor-binding motif 228G [20]. Additionally, every isolate exhibited the H183N and A190V/T substitutions in the receptor-binding site (RBS), which are linked to increased binding affinity for human-like receptors and enhanced replication in mice [21,22]. Notably, amino acid diversity was observed at residue 193 across ten strains, suggesting that this site in H9N2 AIVs from Chinese poultry has undergone frequent mutations since 2013, with N being replaced by A, D, E, G, S, among others [23]. All of these isolates exhibited amino acid deletions in the stalk region (62–64) of the NA protein, associated with virulence in mice. However, none of the isolates carried the E119V, H274Y, or R292K mutations in NA, indicating retained susceptibility to neuraminidase inhibitors [24]. Glycosylation has been increasingly recognized as a key modulator of AIV antigenicity and pathogenicity. Identification of potential glycosylation sites (N-X-S/T motifs, where X represents any amino acid except proline) represents a useful strategy for preventing AIVs [25,26]. Using the NetNGlyc 1.0 Server, five highly conserved potential glycosylation sites (11, 123, 280, 287, and 295) were predicted in the HA1 subunit across all viruses. Six potential glycosylation sites were identified in the NA gene of nine isolates. An N365S substitution in the NA gene of JM14 resulted in the loss of a glycosylation site at this position. Whether alterations at this site affect viral phenotypes warrants further investigation.

Ribonucleoprotein complexes (RNPs) of AIVs play essential roles in the viral infection cycle by regulating RNA replication and transcription. Several mutations known to influence polymerase activity, viral replication, and mammalian adaptation, such as S155N, T271A, K526R, G590S, Q591R, E627K, D701N, and S714R in PB2; K577E in PB1; D347G in PA; and E343K in NP, were not detected in the isolates examined here [12,27,28,29,30,31]. However, several other substitutions were observed, including L89V, I292V, and I588V/I in PB2; I368V and D622G in PB1; 32T, K356R (except FS22), S409N, and 550L in PA; and K398Q in NP, which have been associated with enhanced polymerase activity and viral replication in mammals [32,33,34,35,36,37].

The M2 protein functions as an ion channel in influenza viruses, and mutations in its transmembrane domain can confer resistance to amantadine. All isolates harbored the S31N mutation in M2, indicating resistance to amantadine [17]. In addition, P42S and E92D mutations were identified in the NS1 protein across all ten viruses, which are known to enhance viral resistance to cytokine-mediated antiviral responses [17].

### 3.3. Pathogenicity and Transmission of H9N2 Isolates in Chickens

To assess the pathogenicity and transmissibility of the newly isolated H9N2 viruses in chickens, two strains (JM14 and FS22) from distinct clades of the h9.4.2.5 lineage, as determined by HA phylogenetic analysis, were selected for comparative evaluation of viral replication and transmission.

**Figure 3:**
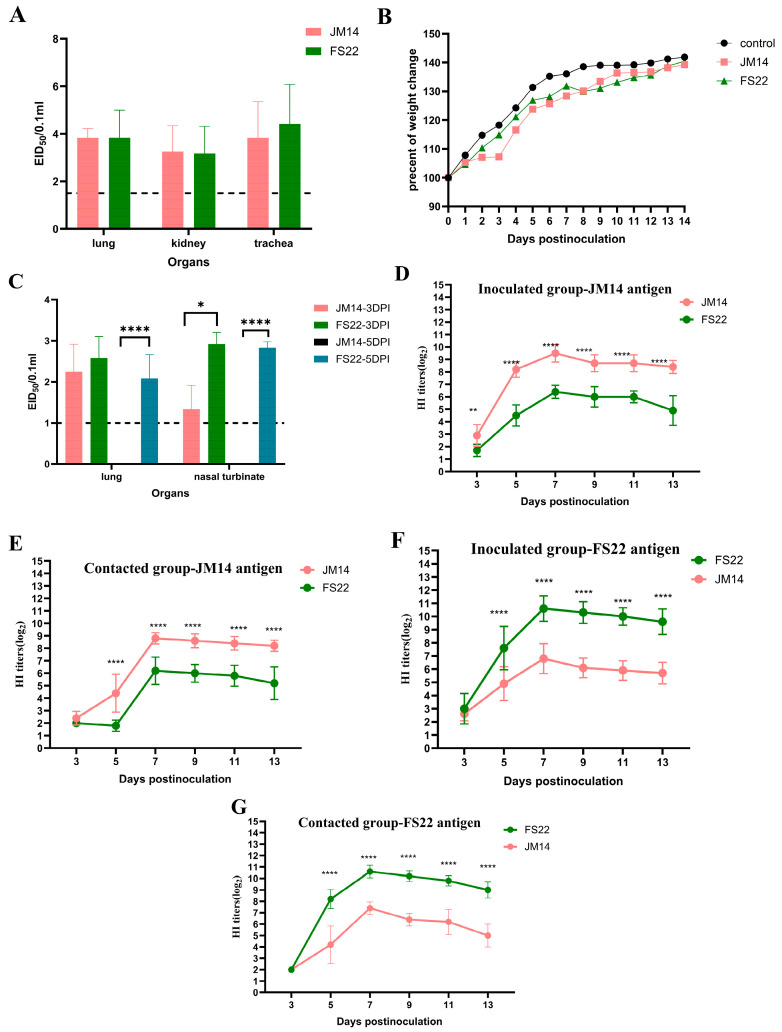
Replication, humoral immunity, and weight change in the H9N2 viruses in chickens and mice. (**A**) Viral titers in organs of SPF chickens. For statistical analysis, a value of 1.5 was assigned if the virus was not detected from the undiluted sample in three embryonated hen eggs [17,18]. (**B**) Weight change in BALB/c mice during the 14 DPI. (**C**) Viral titers in organs of SPF BALB/c mice. For statistical analysis, a value of 1.0 was assigned if the virus was not detected from the undiluted sample in three embryonated hen eggs [17,18]. (**D**–**G**) Cross-HI analysis of chicken antisera. Antibody titers were determined by HI assay using JM14 (**D**,**E**) or FS22 (**F**,**G**) as antigen. For each antigen, sera from homologous and heterologous virus-infected chickens were tested. Data shown were the mean virus titers (log_10_ EID_50_/0.1 mL) ± standard deviation. Statistical analysis was performed using a two-way ANOVA, * *p* < 0.05, ** *p* < 0.01,**** *p* < 0.0001.

Following inoculation, none of the chickens in either group exhibited overt clinical signs. Both JM14 and FS22 viruses replicated efficiently in the lungs, kidneys, and trachea of inoculated chickens. Mean viral titers for JM14 in these tissues were 3.83 ± 0.38, 3.25 ± 1.09, and 3.83 ± 1.53 log_10_ EID_50_/0.1 mL, respectively, while titers for FS22 were 3.83 ± 1.16, 3.17 ± 1.16, and 4.42 ± 1.67 log_10_ EID_50_/0.1 mL. No virus was detected in the hearts, livers, spleens, or brains (Figure 3A).

Viral shedding was monitored by RT-PCR targeting the HA gene in oropharyngeal and cloacal swabs. JM14 was detected exclusively in oropharyngeal swabs at 1 and 3 DPI, with 3/13 and 5/13 birds testing positive, respectively. In contrast, FS22 was shed via both the oropharyngeal and cloacal routes. All inoculated chickens (13/13) shed virus in oropharyngeal swabs at 1 and 3 DPI, with 2/10 remaining positive at 5 DPI. Low-level cloacal shedding of FS22 was also observed in 1/13 and 1/10 birds at 3 and 5 DPI, respectively (Table 1).

To evaluate transmission, contact chickens were introduced. No clinical signs were observed in any contact animals throughout this study. In the JM14 group, 2/5 contact chickens shed virus in oropharyngeal swabs at 5 DPI only. In the FS22 group, viral shedding was detected in contact chickens as early as 1 DPI and persisted through 5 DPI via the oropharyngeal route, with one bird (1/5) also showing cloacal shedding at 3 DPI (Table 1).

To assess the humoral immune response and antigenic relationship, serum samples from inoculated and contact chickens were tested by HI test against homologous and heterologous viral antigens (Figure 3D–G). All chicken groups developed detectable antibodies by 5 DPI, with peak homologous HI titers occurring at 7 DPI (JM14: up to 9.5 ± 0.71 log_2_; FS22: up to 10.6 ± 0.97 log_2_). However, cross-HI tests demonstrated a significant antigenic difference between JM14 and FS22. HI titers of JM14 antiserum against FS22 antigen were significantly lower (1.7 ± 0.48 log_2_ to 6.4 ± 0.52 log_2_), as were FS22 antiserum titers against JM14 antigen (2.0 ± 0 log_2_ to 7.4 ± 0.55 log_2_). The serological results directly mirrored the genetic divergence observed in the HA gene phylogeny.

In summary, both viruses replicated efficiently in chickens and induced a robust humoral immune response rapidly after infection. And the two viruses differed in the duration and routes of viral shedding.

### 3.4. Pathogenicity Studies in Mice

To evaluate the potential mammalian threat posed by these H9N2 AIVs, female SPF BALB/c mice were inoculated with JM14 or FS22. Mice in the control group remained asymptomatic and gained weight throughout the observation period. Neither JM14 nor FS22 inoculated mice exhibited obvious clinical signs. Mice infected with JM14 showed continuous weight gain until the end of the experiment, whereas those inoculated with FS22 displayed transient weight loss at 8 DPI, followed by recovery (Figure 3B).

Viral replication was confined to the lungs and nasal turbinates; no virus was detected in other organs (Figure 3C). JM14 was detected only at 3 DPI in the lungs (2.25 ± 0.67 log_10_ EID_50_/0.1 mL) and nasal turbinates (1.33 ± 0.58 log_10_ EID_50_/0.1 mL). In contrast, FS22 replicated in both tissues at 3 (2.58 ± 0.52 and 2.92 ± 0.30 log_10_ EID_50_/0.1 mL) and 5 DPI (2.08 ± 0.58 and 2.83 ± 0.14 log_10_ EID_50_/0.1 mL), indicating more sustained replication.

These findings demonstrate that both H9N2 isolates are capable of replication in mice without prior adaptation, suggesting a potential capacity for cross-species infection and underscoring their relevance to public health risk assessment.

## 4. Discussion

The persistent circulation and evolution of H9N2 AIV in poultry constitutes a significant challenge to both veterinary and public health. Globally, H9N2 AIVs are categorized into two major lineages: the American lineage (encompassing h9.1 and h9.2 lineages) and the Eurasian lineage (comprising h9.3 and h9.4 lineages) [38]. The h9.4 lineage, particularly the h9.4.2 sub-lineage, has become predominant across most Asian countries since the 1990s. In China, the dominant sub-lineages have shifted over time, from h9.4.2.1–h9.4.2.4 prior to 2007 to the contemporary h9.4.2.5, which has prevailed under ongoing selection pressure since approximately 2010 [1]. This continuous antigenic drift underscores the necessity for timely genetic and phenotypic characterization of circulating strains.

Our study confirms that all ten H9N2 isolates from 2022–2023 belong to the currently dominant h9.4.2.5 lineage. However, detailed phylogenetic analysis revealed that their HA genes segregate into distinct antigenic groups. A critical finding is the significant genetic distance (87.1–93.5% nucleotide identity) between these isolates and the HA genes of current vaccine strains. Effective vaccine-induced protection is contingent upon a close antigenic match between the vaccine and circulating strains. The substantial divergence observed here suggests that the efficacy of existing vaccines against these newly emerged viruses may be suboptimal, potentially facilitating their silent spread in vaccinated flocks. Therefore, empirical evaluation of vaccine efficacy against these contemporary isolates is urgently warranted.

The emergence of novel AIVs is profoundly influenced by genetic reassortment. In China, a confluence of factors, including the co-circulation of multiple influenza virus subtypes in terrestrial poultry, high poultry population density, mixed farming practices, and the existence of live poultry markets, provide favorable conditions for reassortment among different AIV subtypes [39]. Our phylogenetic findings illustrate this dynamic: the internal genes of the new isolates originated from diverse sources, including co-circulating H3, H6, and H9 viruses, revealing a highly active and complex reassortment landscape. Since co-infection of a host with distinct viruses is a prerequisite for reassortment, the high frequency of such events increases the probability of generating novel, transmissible, and potentially pathogenic reassortants. Consequently, implementing biosecurity measures to minimize co-infections is fundamental to reducing the risk of novel virus emergence.

The biological characterization of two representative strains, JM14 and FS22, directly linked their genetic differences to distinct phenotypic outcomes. Despite similar replication profiles in chickens at 3 DPI, the two viruses exhibited distinct shedding profiles and tissue tropism. FS22 exhibited markedly different shedding and transmission dynamics. Its ability to be shed via both respiratory and cloacal routes, coupled with earlier and more efficient transmission to contacts, indicates broader tissue tropism and a higher potential for environmental dissemination, possibly via the fecal-oral route. In contrast, JM14 demonstrated a more restricted, primarily respiratory pattern. The robust HI antibody response confirmed successful infection and seroconversion in all groups. Notably, significant antigenic differences between JM14 and FS22 were revealed by cross-HI tests, highlighting the emergence of antigenic heterogeneity within the h9.4.2.5 lineage. Such heterogeneity complicates vaccine strain selection and may challenge the accuracy of diagnostic strategies. In mice, both viruses replicated without prior adaptation, confirming their inherent potential for cross-species infection. The more sustained replication of FS22 in lungs and nasal turbinates at 3 and 5 DPI, accompanied by transient weight loss, suggests it possesses greater virulence and adaptive potential in mammals compared to JM14. This phenotypic divergence between two viruses from the same major lineage but different subclades underscores that significant functional differences can arise from their specific genetic constellations, likely driven by reassortment. The results of the molecular characteristic analysis indicated that these two viruses possessed a constellation of markers associated with increasing affinity for human-like receptors, such as Q226L, H183N, A190V/T substitutions in RBS of HA. They also had several mutations in other genes (e.g., L89V and I292V in PB2; I368V in PB1; K398Q in NP; P42S, E92D in NS), which were known to enhance polymerase activity in mammalian cells and counteract host antiviral responses. Interestingly, we noticed that these two viruses had different mutations in HA (155N/T; 193T/G), NA (365S/N), PB2 (588I/V), PB1 (577M/K), and PA (356R/K). Whether these mutations were the key factors determining the differences in biological characteristics of the two viruses in chickens and mice requires further study.

## 5. Conclusions

In summary, our integrated genetic and pathological profiling reveals that the ongoing evolution of the h9.4.2.5 H9N2 lineage in China is driving increased genetic and phenotypic diversification. The emergence of strains with attributes such as enhanced mammalian replication and multi-route transmissibility—coupled with significant divergence from vaccine strains—signals an elevated risk potential. This evolving threat landscape calls for robust surveillance systems that synergize genetic and antigenic data, which is paramount for developing updated vaccines and preemptive countermeasures to protect both poultry economies and public health.

## Figures and Tables

**Table 1:** Viral shedding in cloacal and oropharyngeal swabs from inoculated and contacted chickens.

Virus		Virus Shedding on the Days Post-Inoculation (DPI)									
		1		3		5		7		9	
		O ^a^	C ^b^	O	C	O	C	O	C	O	C
JM14	Inoculated chicken	3/13 ^c^	0/13	5/13	0/13	0/10	0/10	0/10	0/10	0/10	0/10
	Contacted chicken	0/5	0/5	2/5	0/5	0/5	0/5	0/5	0/5	0/5	0/5
FS22	Inoculated chicken	13/13	0/13	13/13	1/13	2/10	1/10	0/10	0/10	0/10	0/10
	Contacted chicken	1/5	0/5	5/5	1/5	2/5	0/5	0/5	0/5	0/5	0/5

^a^ oropharyngeal swabs; ^b^ cloacal swabs; ^c^ Number of chickens shedding virus/number of chickens tested.

## Data Availability

The original contributions presented in this study are included in this article. Further inquiries can be directed to the corresponding authors.

## Supplementary Materials

The following supporting information can be downloaded at: https://www.mdpi.com/article/10.3390/microorganisms14010037/s1. Table S1. 10 H9N2 AIVs isolated in the farms of Guangdong, Jiangsu and Shandong provinces during 2022–2023; Table S2. Comparative analysis of HA gene sequence similarity among H9N2 AIV isolates, vaccines, and reference strains; Table S3. Comparative analysis of NA gene sequence similarity among H9N2 AIV isolates, vaccines, and reference strains; Table S4 Influenza viruses with highest nucleotide homology to each gene of ten H9N2 viruses as determined by BLAST search in the GenBank; Table S5. Comparative analysis of key sites of HA1 gene among ten H9N2 viruses and vaccine strains as well as representative strains of different evolutionary branches; Table S6. Molecular characteristics analysis of NA gene of ten H9N2 viruses; Table S7. Molecular characteristics analysis of key amino acid sites in the internal genes of ten H9N2 viruses.

## Abbreviations

The following abbreviations are used in this manuscript:

AIV	Avian influenza virus
DPI	Days post-infection
LPAIV	Low pathogenic avian influenza virus
WHO	World Health Organization
SPF	Specific-pathogen-free
BSL2	Biosafety level 2
IVDC	China Institute of Veterinary Drug Control
RT-PCR	Reverse-transcription polymerase-chain reaction
HI	Hemagglutination inhibition
PBS	Phosphate-buffered saline
HA	Hemagglutination
NA	Neuraminidase
PB2	Polymerase basic subunit 2
PB1	Polymerase basic subunit 1
PA	Polymerase acidic subunit
NP	Nucleoprotein
M	Matrix
NS	Nonstructural
RBS	Receptor-binding site
RNPs	Ribonucleoprotein complexes
