# Supplementary material for: Phylogenetic Analyses and Biological Characterization of H9N2 Avian Influenza Virus Isolated from Chickens in China from 2022 to 2023
Source: Microorganisms. 2025 Dec 23;14(1):37. doi: 10.3390/microorganisms14010037 (PMC12843895; doi:10.3390/microorganisms14010037)
Supplement: Supplementary file 1 [file microorganisms-14-00037-s001.zip › microorganisms-4045764-supplementary.pdf]

Table S1. 10 H9N2 AIVs isolated in the farms of Guangdong, Jiangsu and Shandong provinces during 2022-2023

Virus name	City	Sampling time	GenBank accession number							
			PB2	PB1	PA	HA	NP	NA	M	NS
A/chicken/Guangdong/FS22/2022 (H9N2)	Guangdong	11/2022	PX533972	PX533973	PX533974	PX533975	PX533976	PX533977	PX533978	PX533979
A/chicken/Guangdong/FS08/2023 (H9N2)	Guangdong	3/2023	PX533536	PX533537	PX533538	PX533539	PX533540	PX533541	PX533542	PX533543
A/chicken/Guangdong/JM14/2023 (H9N2)	Guangdong	3/2023	PX533639	PX533640	PX533641	PX533642	PX533643	PX533644	PX533645	PX533646
A/chicken/Guangdong/JM61/2023 (H9N2)	Guangdong	3/2023	PX547946	PX547947	PX547948	PX547949	PX547950	PX547951	PX547952	PX547953
A/chicken/Jiangsu/CZ02/2023 (H9N2)	Jiangsu	3/2023	PX533983	PX533984	PX533985	PX533986	PX533987	PX533988	PX533989	PX533990
A/chicken/Jiangsu/XZ28/2023 (H9N2)	Jiangsu	4/2023	PX548031	PX548032	PX548033	PX548034	PX548035	PX548036	PX548037	PX548038
A/chicken/Jiangsu/XZ30/2023 (H9N2)	Jiangsu	4/2023	PX548039	PX548040	PX548041	PX548042	PX548043	PX548044	PX548045	PX548046
A/chicken/Jiangsu/XZ39/2023 (H9N2)	Jiangsu	5/2023	PX548047	PX548048	PX548049	PX548050	PX548051	PX548052	PX548053	PX548054
A/chicken/Shandong/L392/2023 (H9N2)	Shandong	3/2023	PX547935	PX547936	PX547937	PX547938	PX547939	PX547940	PX547941	PX547942
A/chicken/Shandong/L401/2023 (H9N2)	Shandong	4/2023	PX547885	PX547886	PX547887	PX547888	PX547889	PX547890	PX547891	PX547892

Table S2. Comparative analysis of HA gene sequence similarity among H9N2 AIV isolates, vaccines, and reference strains.

Group	Virus	Nucleotide sequence	Amino acid sequence	Accession number
Isolates in this study	Isolates[a]	91.1%~99.9%	92.2%~99.8%	PX533539- PX548050
Vaccine strains	SS/94[b]	87.3%~88.1%	87.0%~89.0%	AF384557.1
	F/98[c]	86.6%~87.4%	86.8%~89.5%	AY743216.1
	WJ57[d]	92.9%~93.5%	91.6%~94.6%	KJ000710.1
	LG1[e]	92.4%~92.8%	91.4%~94.5%	KC951122.1
	SD6/96[f]	87.1%~87.8%	87.0%~89.7%	DQ064376.1
Reference strains	BJ/94[g]	87.0%~87.8%	86.8%~89.5%	AF156380.1
	Y280[h]	87.2%~88.3%	87.5%~90.7%	AF156376.1
	G1[i]	84.2%~85.6%	85.1%~88.0%	AF156378.1
	G9[j]	86.8%~87.5%	87.3%~90.3%	AF156373.1
	Y439[k]	79.1%~80.2%	82.7%~85.5%	AF156377.1
	Cal/189/66[l]	78.4%~79.3%	83.4%~86.0%	AF156390.1

[a]Isolates: A/chicken/Guangdong/FS22/2022(H9N2): FS22; A/chicken/Guangdong/FS08/2023(H9N2): FS08; A/chicken/Guangdong/JM14/2023(H9N2): JM14; A/chicken/Guangdong/JM61/2023 (H9N2): JM61; A/chicken/Jiangsu/CZ02/2023(H9N2): CZ02; A/chicken/Jiangsu/XZ28/2023 (H9N2): XZ28; A/chicken/Jiangsu/XZ30/2023 (H9N2): XZ30; A/chicken/Jiangsu/XZ39/2023(H9N2): XZ39; A/chicken/Shandong/L392/2023(H9N2): L392; A/chicken/Shandong/L401/2023(H9N2): L401.

[b]A/Chicken/Guangdong/SS/1994(H9N2):SS/94;

[c]A/Chicken/Shanghai/F/1998(H9N2):F/98;

[d]A/chicken/Jiangsu/WJ57/2012(H9N2):WJ57;

[e]A/chicken/Guangdong/LG1/2013(H9N2):LG1;

[f]A/chicken/Shandong/6/1996(H9N2): SD6/96;

[g]A/Chicken/Beijing/1/1994(H9N2): BJ/94;

[h]A/Duck/Hong Kong/Y280/1997(H9N2):Y280;

[i]A/Quail/Hong Kong/G1/1997(H9N2):G1;

[j]A/Chicken/Hong Kong/G9/1997(H9N2):G9;

[k]A/Duck/Hong Kong/Y439/1997(H9N2):Y439; A/turkey/California/189/1966(H9N2): Cal/189/66.

Table S3. Comparative analysis of NA gene sequence similarity among H9N2 AIV isolates, vaccines, and reference strains.

Groups	Virus	Nucleotide sequence	Amino acid sequence	Accession number
Group 1	JM14	96.2%~99.7%	96.6%~99.6%	PX533644- PX547940
	CZ02			
	L392			
	L401			
Group 2	FS22	94.7%~99.7%	93.6%~99.4%	PX533541- PX548052
	XZ39			
	JM61			
	XZ28			
	XZ30			
	FS08			
Vaccine strains	SS/94	88.6%~90.0%	88.6%~89.9%	DQ874395.1
	F/98	87.8%~89.0%	89.7%~91.0%	AY253754.1
	WJ57	89.4%~91.2%	90.3%~91.2%	KP893706.1
	LG1	92.2%~93.6%	92.1%~94.0%	KC951124.1
	SD6/96	88.2%~89.5%	88.6%~90.1%	DQ064430.1
Reference strains	BJ/94	87.8%~89.4%	88.2%~89.5%	AF156398.1
	Y280	88.2%~89.6%	89.1%~90.3%	AF156394.1
	G1	85.6%~87.2%	86.3%~87.8%	AF156396.1
	G9	86.4%~87.7%	86.7%~87.8%	AF156391.1
	Y439	83.6%~84.8%	82.8%~85.0%	AF156395.1
	Cal/189/66	82.1%~82.9%	83.2%~84.7%	AF156401.1

Table S4 Influenza viruses with highest nucleotide homology to each gene of ten H9N2 viruses as determined by BLAST search in the GenBank

strains	Gene [a]	Closest viruses in GenBank	Homolog Nucleotide（%）
A/chicken/Guangdong/FS22/2022	PB2	A/chicken/Guangzhou/199/2022(H3N8)	99.34
(H9N2)	PB1	A/chicken/Guangzhou/4463/2021(H3N8)	99.60
	PA	A/chicken/Hong Kong/21-17632/2021(H3N8)	99.63
	HA	A/chicken/China/GD2021/2021(H9N2)	97.92
	NP	A/chicken/Anhui/CKAH02/2022(H3N8)	98.80
	NA	A/chicken/China/YL22/2022(H9N2)	99.07
	M	A/chicken/Shanxi/12.12_JZRL-O24/2021(H9N2)	98.57
	NS	A/chicken/Fujian/11.18_FZHX61-O/2019(H9N2)	98.88
	PB2	A/chicken/China/Guangdong_01/2022(H6N8)	98.82
A/chicken/Guangdong/FS08/2023	PB1	A/chicken/Anhui/05.31ZGS11-O/2021(H9N2)	98.90
(H9N2)	PA	A/chicken/Anhui/05.31ZGS14-O/2021(H9N2)	98.88
	HA	A/chicken/China/GD2021/2021(H9N2)	97.56
	NP	A/chicken/Fujian/12.26FZHX2112-137-O/2021(H9N2)	98.86
	NA	A/pigeon/Fujian/12.22_FZHX97-C/2020(H9N2)	96.15
	M	A/Duck/Guangdong/FS91/2022(H9N2)	98.68
	NS	A/chicken/Jiangxi/10.31_NCDGL007-O/2018(H9N2)	98.76
	PB2	A/silkie chicken/Shantou/4181/2021(H3N8)	98.16
A/chicken/Guangdong/JM14/2023	PB1	A/Duck/Guangdong/FS91/2022(H9N2)	98.37
(H9N2)	PA	A/chicken/China/YL22/2022(H9N2)	98.74
	HA	A/environment/Xiamen/01/2021(H9N2)	98.24
	NP	A/chicken/Fujian/12.26FZHX2112-142-O/2021(H9N2)	98.60
	NA	A/chicken/Zhejiang/7.23_HZBX002-O/2018(H9N2)	97.86
	M	A/duck/China/9-12/2022(H6N6)	98.88
	NS	A/chicken/Fujian/6.29FZHX0710-178-2-C/2021(H9N2)	99.33
A/chicken/Guangdong/JM61/2023	PB2	A/chicken/China/HN0120/2023(H3N3)	99.47
(H9N2)	PB1	A/chicken/Shangqiu/SQ2023/2023(H9N2)	99.21
	PA	A/China/ZMD-22-2/2022(H3N8)	99.58
	HA	A/chicken/Shangqiu/SQ2023/2023(H9N2)	98.63
	NP	A/China/ZMD-22-2/2022(H3N8)	99.53
	NA	A/chicken/China/YL22/2022(H9N2)	98.93
	M	A/China/ZMD-22-2/2022(H3N8)	99.59
	NS	A/chicken/Shangqiu/SQ2023/2023(H9N2)	99.55
A/chicken/Jiangsu/CZ02/2023	PB2	A/chicken/China/YC01/2023(H3N3)	99.69
(H9N2)	PB1	A/chicken/Jiangsu/CKJS03/2022(H3N8)	99.08
	PA	A/chicken/Jiangxi/8.30NCJD29-O/2022(H9N2)	99.49
	HA	A/chicken/Fujian/12.26FZHX2112-178-C/2021(H9N2)	98.54
	NP	A/chicken/Jiangxi/7.25NCJD13-O/2022(H9N2)	99.60
	NA	A/chicken/Shandong/049/2020(H9N2)	97.93
	M	A/chicken/China/16/2023(H3N3)	99.49
	NS	A/chicken/China/G1451/2016(H9N2)	98.76
A/chicken/Jiangsu/XZ28/2023	PB2	A/chicken/China/HN0120/2023(H3N3)	99.69
(H9N2)	PB1	A/chicken/Shangqiu/SQ2023/2023(H9N2)	99.04
	PA	A/China/ZMD-22-2/2022(H3N8)	99.63
	HA	A/chicken/Shangqiu/SQ2023/2023(H9N2)	98.64

	NP	A/China/ZMD-22-2/2022(H3N8)	99.47
	NA	A/chicken/China/YL22/2022(H9N2)	98.79
	M	A/China/ZMD-22-2/2022(H3N8)	99.39
	NS	A/chicken/Shangqiu/SQ2023/2023(H9N2)	99.55
A/chicken/Jiangsu/XZ30/2023 (H9N2)	PB2	A/Duck/Guangdong/FS91/2022(H9N2)	99.21
	PB1	A/chicken/Shandong/65/2021(H9N2)	98.11
	PA	A/chicken/Viet Nam/LBOral150LS/2022(H9N2)	99.22
	HA	A/chicken/Viet Nam/LBOral150LS/2022(H9N2)	99.11
	NP	A/chicken/China/YL21/2022(H9N2)	98.73
	NA	A/chicken/Viet Nam/LBOral150LS/2022(H9N2)	99.22
	M	A/chicken/Henan/11.24XXWJX12-O/2021(H9N2)	99.78
	NS	A/chicken/Fujian/6.29FZHX0710-174-2-C/2021(H9N2)	98.09
A/chicken/Jiangsu/XZ39/2023 (H9N2)	PB2	A/chicken/China/HN0120/2023(H3N3)	99.39
	PB1	A/chicken/Shangqiu/SQ2023/2023(H9N2)	98.99
	PA	A/China/ZMD-22-2/2022(H3N8)	99.59
	HA	A/chicken/Shangqiu/SQ2023/2023(H9N2)	98.76
	NP	A/China/ZMD-22-2/2022(H3N8)	99.47
	NA	A/chicken/China/YL22/2022(H9N2)	98.79
	M	A/China/ZMD-22-2/2022(H3N8)	99.69
	NS	A/chicken/Shangqiu/SQ2023/2023(H9N2)	99.33
A/chicken/Shandong/L392/2023 (H9N2)	PB2	A/chicken/China/YC01/2023(H3N3)	99.47
	PB1	A/chicken/China/16/2023(H3N3)	99.16
	PA	A/chicken/China/YL22/2022(H9N2)	98.14
	HA	A/chicken/Fujian/12.26FZHX2112-178-C/2021(H9N2)	98.30
	NP	A/chicken/China/16/2023(H3N3)	99.60
	NA	A/chicken/Shandong/049/2020(H9N2)	98.00
	M	A/chicken/China/16/2023(H3N3)	99.59
	NS	A/chicken/Shandong/3.25TAFC2-O/2021(H9N2)	98.65
A/chicken/Shandong/L401/2023 (H9N2)	PB2	A/chicken/China/YC01/2023(H3N3)	99.43
	PB1	A/chicken/Jiangsu/CKJS03/2022(H3N8)	98.90
	PA	A/chicken/Jiangxi/8.30NCJD29-O/2022(H9N2)	99.40
	HA	A/duck/Fujian/9.27FZHX2110-158-C/2021(H9N2)	98.81
	NP	A/chicken/Jiangxi/7.25NCJD13-O/2022(H9N2)	99.73
	NA	A/chicken/Shandong/049/2020(H9N2)	97.93
	M	A/chicken/China/16/2023(H3N3)	99.29
	NS	A/chicken/China/G1451/2016(H9N2)	98.88

[a]PB2, polymerase basic 2; PB1, polymerase basic 1; PA, polymerase acidic; NP, nucleoprotein; NA, neuraminidase; M, matrix; NS, nonstructural.

Table S5. Comparative analysis of key sites of HA1 gene among ten H9N2 viruses and vaccine strains as well as representative strains of different evolutionary branches

Virus	receptor binding sites[a]									potential glycosylation sites[b]						cleavage site
	155	158	183	187	190	193	226	227	228	11-13	87-89	123-125	280-282	287-289	295-297	
FS22	T	N	N	T	T	G	L	M	G	NST	/	NVS	NTT	NVS	NCS	PSRSSR↓GLF
FS08	T	N	N	T	T	G	M	M	G	NST	/[c]	NVS	NTT	NVS	NCS	PSRSSR↓GLF
JM14	N	N	N	T	V	T	L	M	G	NST	/	NVS	NTT	NVS	NCS	PSRSSR↓GLF
JM61	N	N	N	T	V	N	L	M	G	NST	/	NVS	NTT	NVS	NCS	PSRSSR↓GLF
CZ02	N	N	N	T	A	T	L	M	G	NST	/	NVS	NTT	NVS	NCS	PSRSSR↓GLF
XZ28	N	N	N	T	V	N	L	M	G	NST	/	NVS	NTT	NVS	NCS	PSRSSR↓GLF
XZ30	N	N	N	T	V	T	L	M	G	NST	/	NVS	NTT	NVS	NCS	PSRSSR↓GLF
XZ39	N	N	N	T	V	N	L	M	G	NST	/	NVS	NTT	NVS	NCS	PSRSSR↓GLF
L392	N	N	N	T	V	T	L	M	G	NST	/	NVS	NTT	NVS	NCS	PSRSSR↓GLF
L401	N	N	N	T	V	T	L	M	G	NST	/	NVS	NTT	NVS	NCS	PSRSSR↓GLF
SS/94	T	N	N	T	A	N	Q	Q	G	NST	/	NVS	NTT	NVS	/	PAGSSR↓GLF
F/98	T	N	N	T	A	N	Q	Q	G	NST	/	NVS	NTT	NVS	/	PARSSR↓GLF
WJ57	T	N	N	T	V	N	L	M	G	NST	/	NVS	NTT	NVS	NCS	PSRSSR↓GLF
LG1	T	N	N	T	V	N	L	Q	G	NST	/	NVS	NTT	NVS	NCS	PSRSSR↓GLF
SD6/96	T	D	N	T	A	N	Q	Q	G	NST	/	NVS	NTT	NVS	/	PARSSR↓GLF
BJ/94	T	N	N	T	V	N	Q	Q	G	NST	/	NVT	NTT	NVS	/	PARSSR↓GLF
Y280	T	N	N	T	T	N	L	Q	G	NST	/	NVS	NTT	NVS	/	PARSSR↓GLF
G1	T	S	H	T	E	N	L	Q	G	NST	NGT	NVT	NST	NIS	/	PARSSR↓GLF
G9	T	N	N	T	A	N	L	Q	G	NST	/	NVS	NTT	NVS	/	PARSSR↓GLF
Y439	T	S	H	T	E	N	Q	Q	G	NST	/	NVT	NTT	NVS	/	PAASNR↓GLF
Cal/189/66	T	S	H	T	E	D	Q	Q	G	NST	/	NVT	NTT	NIS	/	PAVSSR↓GLF

[a] all of HA genes are in H3 number; [b] all of HA genes are in H9 number without signal peptide; [c]Not detected.

Table S6. Molecular characteristics analysis of NA gene of ten H9N2 viruses

Virus[a]	stalk region	Potential glycosylation sites						Drug resistance site		
	62-64 [b]	66-68	83-85	143-145	197-199	231-233	365-367	119	274	292
FS22	Delete	NST	NWS	NGT	NAT	NGT	NSS	E	H	R
FS08	Delete	NST	NWS	NGT	NAT	NGT	NSS	E	H	R
JM14	Delete	NST	NWS	NGT	NAT	NGT	/	E	H	R
JM61	Delete	NST	NWS	NGT	NAT	NGT	NSS	E	H	R
CZ02	Delete	NST	NWS	NGT	NAT	NGT	NGS	E	H	R
XZ28	Delete	NST	NWS	NGT	NAT	NGT	NSS	E	H	R
XZ30	Delete	NNT	NWS	NGT	NAT	NGT	NSS	E	H	R
XZ39	Delete	NST	NWS	NGT	NAT	NGT	NSS	E	H	R
L392	Delete	NST	NWS	NGT	NAT	NGT	NGS	E	H	R
L401	Delete	NST	NWS	NGT	NAT	NGT	NGS	E	H	R

[a]the abbreviations for viruses are shown in Table S2; [b]all of NA genes are in N2 number without signal peptide.

Table S7. Molecular characteristics analysis of key amino acid sites in the internal genes of ten H9N2 viruses

Virus[a]	PB2											PB1			PA						NP		M2	NS1	
	89	155	271	292	526	588	590	591	627	701	714	368	577	622	32	49	347	356	409	550	398	434	31	42	92
FS22	V	S	T	V	K	V	G	Q	E	D	S	V	K	G	T	S	D	K	N	L	Q	E	N	S	D
FS08	V	S	T	V	K	V	G	Q	E	D	S	V	K	G	T	S	D	R	N	L	Q	E	N	S	D
JM14	V	S	T	V	K	I	G	Q	E	D	S	V	M	G	T	S	D	R	N	L	Q	E	N	S	D
JM61	V	S	T	V	K	V	G	Q	E	D	S	V	K	G	T	S	D	R	N	L	Q	E	N	S	D
CZ02	V	S	T	V	K	V	G	Q	E	D	S	V	K	G	T	S	D	R	N	L	Q	E	N	S	D
XZ28	V	S	T	V	K	V	G	Q	E	D	S	V	K	G	T	S	D	R	N	L	Q	E	N	S	D
XZ30	V	S	T	V	K	V	G	Q	E	D	S	V	K	G	T	S	D	R	N	L	Q	E	N	S	D
XZ39	V	S	T	V	K	V	G	Q	E	D	S	V	K	G	T	S	D	R	N	L	Q	E	N	S	D
L392	V	S	T	V	K	V	G	Q	E	D	S	V	K	G	T	S	D	R	N	L	Q	E	N	S	D
L401	V	S	T	V	K	V	G	Q	E	D	S	V	K	G	T	S	D	R	N	L	Q	E	N	S	D

[a]the abbreviations for viruses are shown in Table S2.
